# Multifaceted bioinformatic analysis of m6A‐related ferroptosis and its link with gene signatures and tumour‐infiltrating immune cells in gliomas

**DOI:** 10.1111/jcmm.70060

**Published:** 2024-09-09

**Authors:** Yang Yang, Liu Hao, Liu Guiyang, Piao Haozhe

**Affiliations:** ^1^ Liaoning University of Traditional Chinese Medicine Shenyang Liaoning People's Republic of China; ^2^ TCM Department The First Affiliated Hospital of Dalian Medical University Dalian Liaoning People's Republic of China; ^3^ Department of Neurosurgery The Fourth People's Hospital of Jinan Jinan Shandong People's Republic of China; ^4^ Department of Neurosurgery Liaoning Cancer Hospital & Institute Shenyang Liaoning People's Republic of China

**Keywords:** ferroptosis, low‐grade gliomas, m6A modification

## Abstract

Whether N6‐Methyladenosine (m6A)‐ and ferroptosis‐related genes act on immune responses to regulate glioma progression remains unanswered. Data of glioma and corresponding normal brain tissues were fetched from the TCGA database and GTEx. Differentially expressed genes (DEGs) were identified for GO and KEGG enrichment analyses. The FerrDb database was based to yield ferroptosis‐related DEGs. Hub genes were then screened out using the cytoHubba database and validated in clinical samples. Immune cells infiltrating into the glioma tissues were analysed using the CIBERSORT R script. The association of gene signature underlying the m6A‐related ferroptosis with tumour‐infiltrating immune cells and immune checkpoints in low‐grade gliomas was analysed. Of 6298 DEGs enriched in mRNA modifications, 144 were ferroptosis‐related; NFE2L2 and METTL16 showed the strongest positive correlation. METTL16 knockdown inhibited the migrative and invasive abilities of glioma cells and induced ferroptosis in vitro. NFE2L2 was enriched in the anti‐m6A antibody. Moreover, METTL16 knockdown reduced the mRNA stability and level of NFE2L2 (both *p* < 0.05). Proportions of CD8+ T lymphocytes, activated mast cells and M2 macrophages differed between low‐grade gliomas and normal tissues. METTL16 expression was negatively correlated with CD8+ T lymphocytes, while that of NFE2L2 was positively correlated with M2 macrophages and immune checkpoints in low‐grade gliomas. Gene signatures involved in the m6A‐related ferroptosis in gliomas were identified via bioinformatic analyses. NFE2L2 interacted with METTL16 to regulate the immune response in low‐grade gliomas, and both molecules may be novel therapeutic targets for gliomas.

## 1 INTRODUCTION

Gliomas, the most common primary malignancy of the central nervous system, are categorized into four grades based on their malignancy level, as defined by the World Health Organization (WHO).^1^ Low‐grade (I and II) gliomas are characterized by slower growth and less aggressive behaviours. In contrast, high‐grade (III and IV) gliomas, including glioblastomas, are known for their rapid growth and aggressive nature, and have a poor prognosis, with a 5‐year relative survival rate of less than 6.9%.^2^ Therefore, early detection and immediate treatment are essential to prolong the survival of patients diagnosed with gliomas.

Traditional treatment approaches for glioma primarily include surgical intervention, chemotherapy and radiation therapy.^3^ The intricate biological makeup of glioma cells, characterized by their high diversity, rapid proliferation and tendency for infiltration, significantly contributes to their high rates of recurrence and drug resistance.^4^ Despite previous explorations into the pathological mechanisms of glioma, patients' quality of life remains distressingly low.^5^ Consequently, new therapeutic and diagnostic biomarkers with high sensitivity and specificity remain to be discovered.^6^, ^7^, ^8^

Ferroptosis features iron‐dependent accumulation of lipid peroxides.^9^, ^10^ The role of ferroptosis in the development of glioma has been clarified as essential. Ferroptosis‐related gene models have shown excellent performances in predicting the risk of glioma.^11^, ^12^ Therefore, molecules involved in ferroptosis may be targeted to design effective strategy for glioma.

Anti‐cancer therapy based on ferroptosis has been widely explored in recent years, especially in the epigenetic regulations at transcriptional and translational levels.^13^ Accumulating evidence has revealed the potential correlation between N6‐methyladenosine (m6A) modification and ferroptosis.^14^

M6A methylation is among the most prevalent epigenetic modifications found in eukaryotic RNA.^15^ It is a reversible chemical process dynamically controlled by methyltransferases and demethylases.^16^ Current research shows that abnormal m6A modification may result in poor prognosis and chemotherapy resistance in various types of cancers. m6A‐related angiogenesis genes can be used to predict the prognosis of hepatocellular carcinoma (HCC) patients.^17^ FTO promotes the malignancy and chemotherapy resistance of acute myeloid leukaemia by inducing m6A demethylation.^18^ Its methylation‐related factors can act as tumour promoters or suppressors to regulate the processes of glioma cells, and may be new therapeutic targets for glioma.^19^

Tumour cells are like ‘seeds’, which rely on the ‘soil’, the surrounding microenvironment, to grow and spread (metastasis).^20^, ^21^ Tumour cells, along with its adjacent cellular and non‐cellular components, form a tumour microenvironment (TME) responsible for the behaviour and progression of cancer cells.^22^

Increasing studies have teased out an interplay between ferroptosis and tumour immunity.^23^, ^24^ Ferroptosis synergistically enhances the effect of immunotherapy, laying a theoretical basis for designing combination therapies to combat cancers.^25^ Ferroptosis inducers suppress tumour cells, but also change the function and activity of immune cells within the TME.^26^ In addition, m6A modification can regulate the infiltration, survival, differentiation or polarisation of immune molecules in the TME, to control the efficacy of immunotherapy.^27^ The innate immunity exerts a dual role in cancer progression.^28^ It either inhibits cancer cell behaviours by promoting the adaptive tumour‐specific T cell responses through antigen presentation, cytokine support and direct anti‐tumour activity, or promotes cancer cell growth through cytokine release and immune suppression.^29^, ^30^ Biological functions of innate immune system cells can also be altered by ferroptosis and m6A.^31^ We believed that an in‐depth exploration of the interactions between ferroptosis, m6A and anti‐tumour immunity contributes to the development of gliomas‐targeted therapeutic strategies.

The Cancer Genome Atlas (TCGA), a project initially launched in 2005, catalogues cancer‐causing genomic alterations in human beings and thus reveals the mechanisms underlying carcinogenesis and cancer progression.^32^ The Genotype‐Tissue Expression (GTEx) project provides a resource of genetic variants in normal human tissues and organs, which differs from that of the TCGA analysing tumour‐associated sequencing data.^33^ Multi‐phenotype models have become popular in predicting the outcomes of cancer treatments. In this study combining m6A modification, ferroptosis and TME, we aimed to explore new mechanisms and therapeutic targets for resolving gliomas.

## 2 MATERIALS AND METHODS

### 2.1 **Identification** of **DEGs**

Transcriptomic data of 534 low‐grade glioma tissues and 1152 normal brain tissue samples were taken from the TCGA database (https://portal.gdc.cancer.gov) and GTEx project (http://xena.ucsc.edu/), respectively.^34^ Differentially expressed genes (DEGs) were identified through the Limma package in R, according to a fold change (FC) >2 and an adjusted *p* < 0.05.^35^

### 2.2 GO and KEGG analyses

GO terms and significant pathways engaged in these DEGs were analysed and visualized. Both analyses were accomplished using the clusterProfiler, org.Hs.eg.db, and enrichplot packages in R, as previously reported.^36^

### 2.3 Identification of ferroptosis‐related DEGs

Ferroptosis regulators in human glioma tissues were screened in the FerrDb database (http://www.zhounan.org/ferrdb/current/).^37^ Intersections between 6298 DEGs and 296 ferroptosis regulators in human gliomas were visualized by plotting a Venn diagram using the R package.

### 2.4 Search for hub genes and establishment of a protein–protein interaction (PPI) network

The top 10 ferroptosis‐related DEGs between normal and diseased tissues were screened using the cytoHubba 3.7, and their expression levels were measured using the unpaired two‐samples Wilcoxon test in R.^38^ The PPI of hub genes was visualized using the STRING online database, with the confidence score of 0.4 and above.^39^

### 2.5 Identification of tumour‐infiltrating immune cells and correlation analysis

Datasets containing low‐grade glioma tissues from the TCGA database were analysed by the CIBERSORT R script.^40^ Gene expression profiles with a *p* < 0.05 were selected. The abundances of tumour‐infiltrating cells were measured. Correlation matrixes of tumour‐infiltrating immune cells were visualized with the corrplot package in R.

A total of 26 common m6A regulators were selected through literature review.^14^, ^41^, ^42^ The SRAMP (sequence‐based RNA adenosine methylation site predictor) prediction server was adopted to predict mammalian m6A sites.^43^

Associations between hub genes and m6A regulators were measured using the cor.test function in R (|Spearman R| >0.3 and *p* < 0.1) and NFE2L2 was identified as the hub gene with the strongest correlation with the m6A writer METTL16. Their expression levels in glioma tissues were explored in the UALCAN database.^44^

Differences in tumour‐infiltrating immune cells of low‐grade glioma tissues expressing high levels of METTL16 and NFE2L2 versus those expressing low levels were reflected by vioplot package of R.

Correlations of METTL16 or NFE2L2 with tumour‐infiltrating immune cells or immune checkpoints (TNFSF4, PDCD1, CD244 and ICOS) were analysed using the cor.test function in R and an online tool designed for revealing the relationship between a single gene and immune checkpoints (http://www.sxdyc.com/panCancerIcb), respectively.

### 2.6 Culture and transfection

The human glioma cell lines LN229 and A172 (American Type Culture Collection, ATCC) and the human meningeal cell line (HMC) were cultured in the DMEM (BasalMedia, Shanghai, China) containing faecal bovine serum (FBS) (10%) and penicillin–streptomycin solution (1%) in an incubator with 95% air and 5% CO_2_ at 37°C. Transfection of si‐Control or METTL16 siRNAs (siMETTL16‐1 and siMETTL16‐2, GenePharma, Shanghai, China) was performed using the GoldenTran‐R (Golden Transfer Technology, Changchun, China) for 48 h.^45^ Sequences of METTL16 siRNAs were listed as follows: siMETTL16‐1 (sense, CCAUGACAGUCUACAACUUTT; antisense, AAGUUGUAGACUGUCAUGGTT) and siMETTL16‐2 (sense, CCUUGAGACUCAACUAUAUTT; antisense, AUAUAGUUGAGUCUCAAGGTT).

### 2.7 qRT‐PCR

Cellular RNA was collected using TRIzol (ABclonal, Wuhan, China), and reversely transcribed into cDNA using the ABScript Neo RT Master Mix for qPCR with gDNA Remover (ABclonal). Thermal reactions of interested genes were performed using the BrightCycle Universal SYBR Green qPCR Mix with UDG at 37°C for 2 min and 95°C for 3 min, accompanied by 40 cycles of 95°C for 5 s and 60°C for 30 s on the CFX96 Touch™ Real‐Time PCR system (Bio‐Rad, Hercules, CA, USA).^46^ Sequences of primers included: METTL16 (forward, 5′‐TTCTGTCAAGGTCGGACAATG‐3′; reverse, 5′‐CAGCACCACGAATGTTATGGG‐3′), NFE2L2 (forward, 5′‐TTCCCGGTCACATCGAGAG‐3′; reverse, 5′‐TCCTGTTGCATACCGTCTAAATC‐3′); GAPDH (forward, 5′‐ACATCGCTCAGACACCATG‐3′; reverse, 5′‐TGTAGTTGAGGTCAATGAAGGG‐3′). Relative levels were measured by the 2^−ΔΔCT^ method and normalized to that of GAPDH.

### 2.8 Methylated RNA immunoprecipitation and qRT‐PCR (MeRIP‐qPCR)

*MeRIP‐qPCR* was performed with m6A RNA Methylation Quantification Kit (Epibioteck, R1814). Total RNA was fragmented and prepared for magnetic beads, which were then immunoprecipitated, eluted and purified. Purified RNA was finally analysed by qRT‐PCR.^47^

### 2.9 Transwell assay

Transwell chambers with and without pre‐coated diluted Matrigel were taken to detect cell migration and invasion, respectively. Briefly, serum‐free LN229 cells were implanted on the upper compartment of 6‐well plates, with the bottom added with medium containing FBS at 10%. After a 24‐h cell culture, the cells having migrated or invaded to the bottom were fixed. Having been stained with 0.1% crystal violet, the cells were captured through an inverted microscope, and counted.^48^

### 2.10 mRNA stability assay

Transfected cells were implanted into 6‐well plates and cultured until the confluence of 70%. Cells were then induced with actinomycin D 15 μg/mL for 0, 1.5, 2.5 and 3.5 h, followed by mRNA detection by qRT‐PCR.^49^

### 2.11 Measurement of ROS, MDA and GSH

LN229 cells were implanted into 12‐well plates (1 × 10^5^ cells per well), and cultured until attachment. Fresh Hanks' Balanced Salt Solution (HBSS) that contained 10 μM H_2_DCFH‐DA probe (Invitrogen, Carlsbad, CA, USA) was added per well and cultured for 30 min at 37°C. Cell nuclei were stained by Hoechst 33258. Reactive oxygen species (ROS) were observed under a fluorescence microscope.^50^

The contents of malondialdehyde (MDA) and glutathione (GSH) in LN229 cells were measured using commercial kits (Sigma‐Aldrich, St. Louis, USA) according to the manufacturers' recommendations.

### 2.12 Statistical analyses

All cell experiments were conducted in triplicate. Statistical analyses were carried out using SPSS version 25.0 and R version 4.1.3. Associations of m6A RNA methylation regulators with their targets were measured using the Spearman correlation coefficient. Between‐group differences were assessed through the unpaired two‐tailed Student's *t*‐test, while those among multiple groups were evaluated through one‐way ANOVA followed by Tukey's post hoc test. All data were expressed as mean ± SEM. *p* < 0.05 was considered statistically significant, unless stated otherwise. Graphical representations of the data were produced by GraphPad Prism (version 8.0).

## 3 RESULTS

### 3.1 DEGs between glioma and normal brain tissues

**FIGURE 1:**
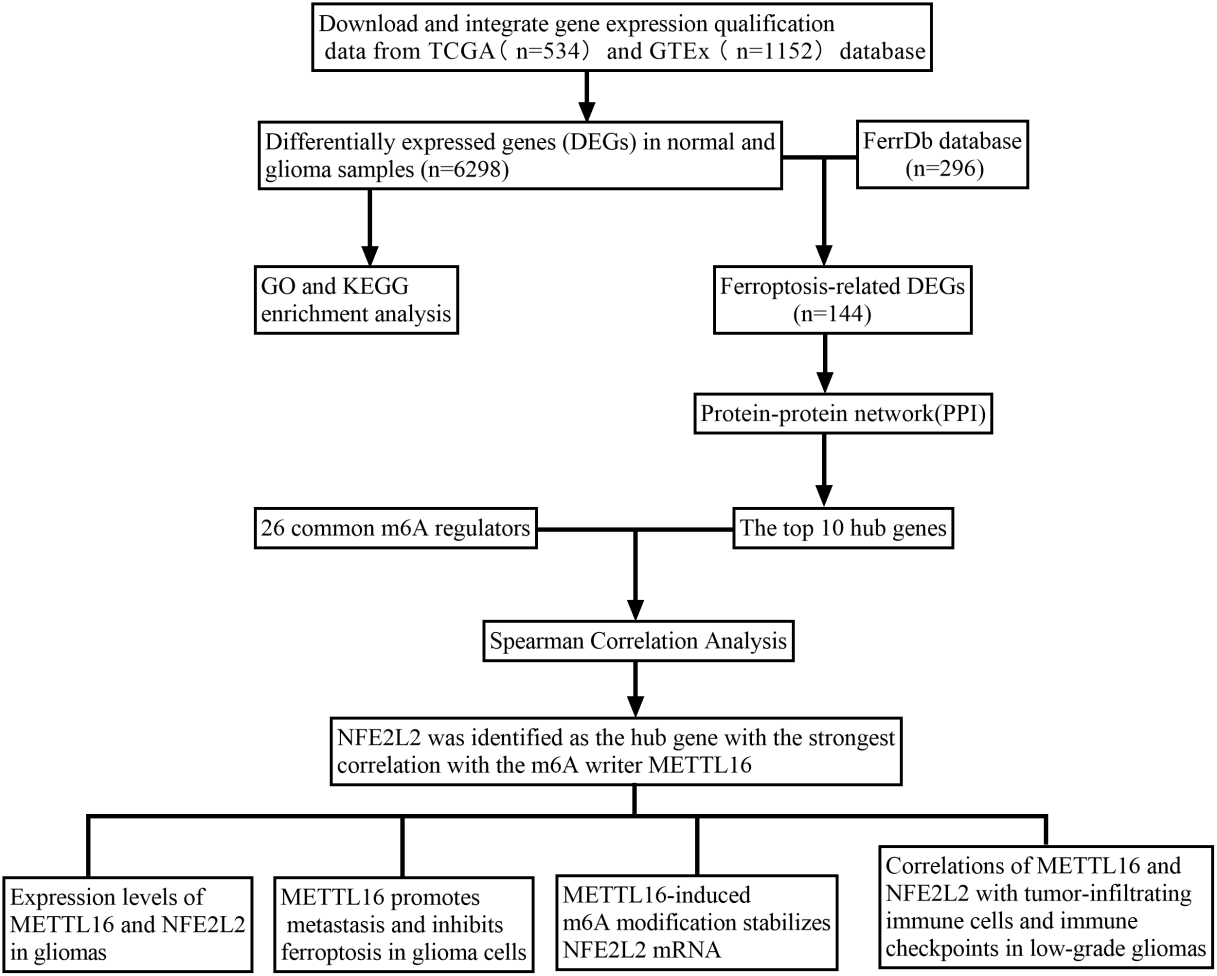
Research design flow chart.

**FIGURE 2:**
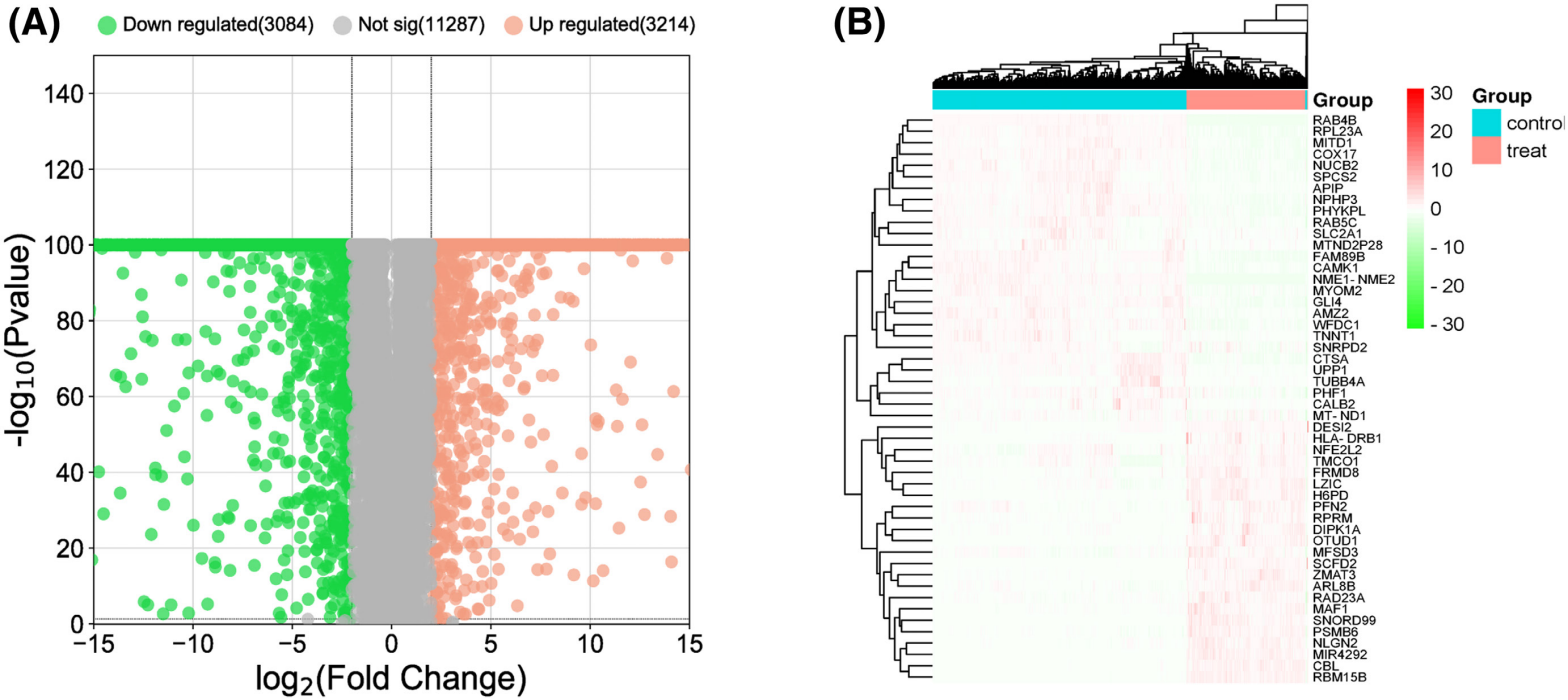
DEGs in the TCGA (*n* = 534) and GTEx (*n* = 1152). (A) Volcanic map of DEGs. Red, up‐regulated; green, down‐regulated. (B) Heat map of DEGs. Red, up‐regulated; green, down‐regulated.

The research flowchart for this study is presented in Figure 1. Data in the glioma tissues (*n* = 534) of the TCGA database and normal brain tissues (*n* = 1152) of the GTEx project were mined. In total, 6298 DEGs were filtered out: 3214 upregulated and 3084 downregulated (Figure 2A,B).

### 3.2 Enrichments of DEGs

**FIGURE 3:**
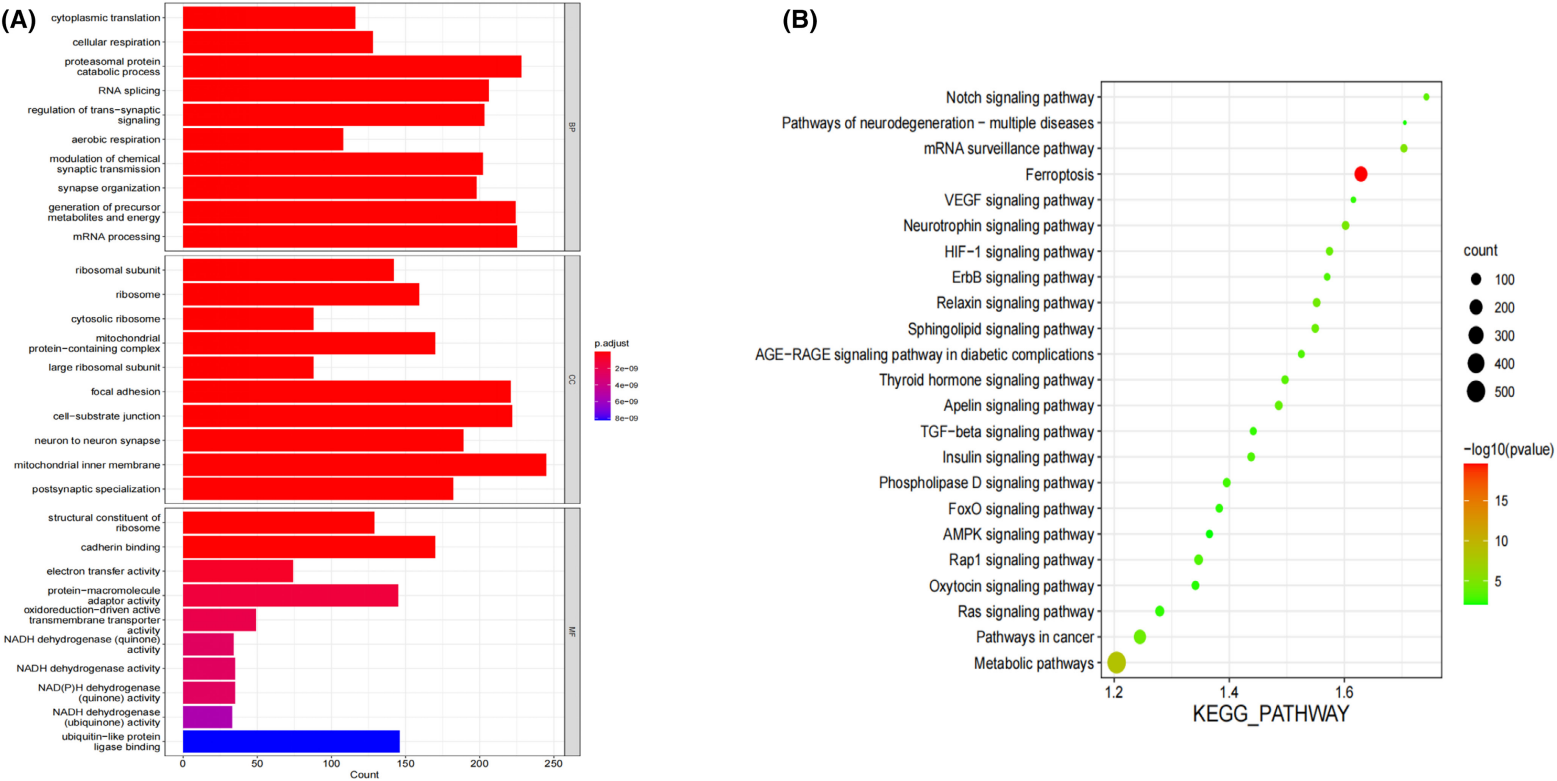
GO (A) and KEGG (B) enrichment analyses of 6298 DEGs between glioma (*n* = 534) and normal brain tissues (*n* = 1152).

The identified 6298 DEGs were subjected to the GO and KEGG enrichment analyses. As shown in Figure 3A, enriched BPs included proteasomal protein catabolic process, generation of precursor metabolites and energy and mRNA processing, MFs of structural constituent of ribosome, cadherin binding and protein‐macromolecule adaptor activity, etc. In addition, they were mainly involved in pathways related with ferroptosis, metabolism and cancer (Figure 3B).

### 3.3 Ferroptosis‐related DEGs and hub genes

**FIGURE 4:**
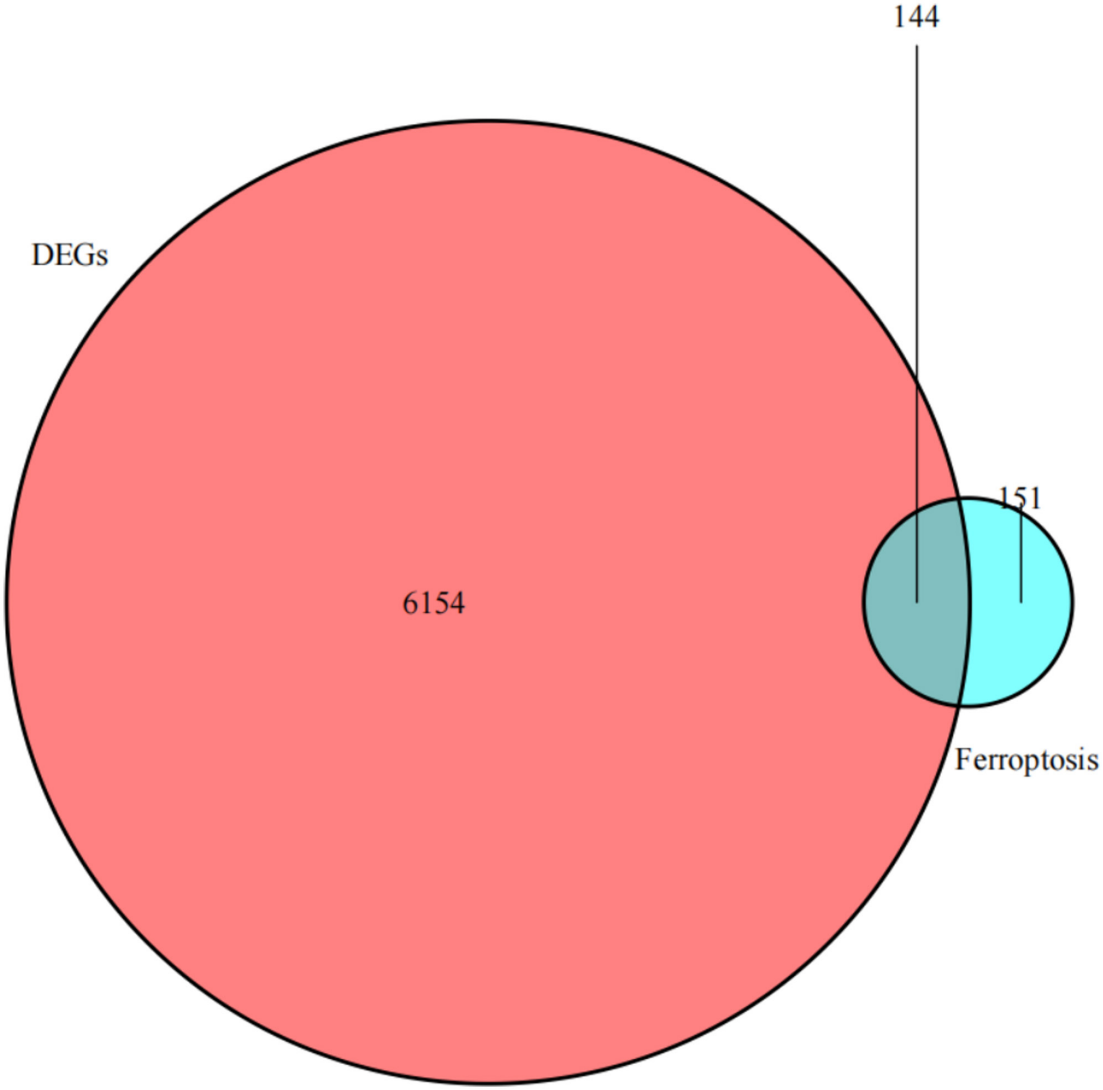
The Venn diagram visualising 144 ferroptosis‐related DEGs between glioma and normal brain tissues.

**FIGURE 5:**
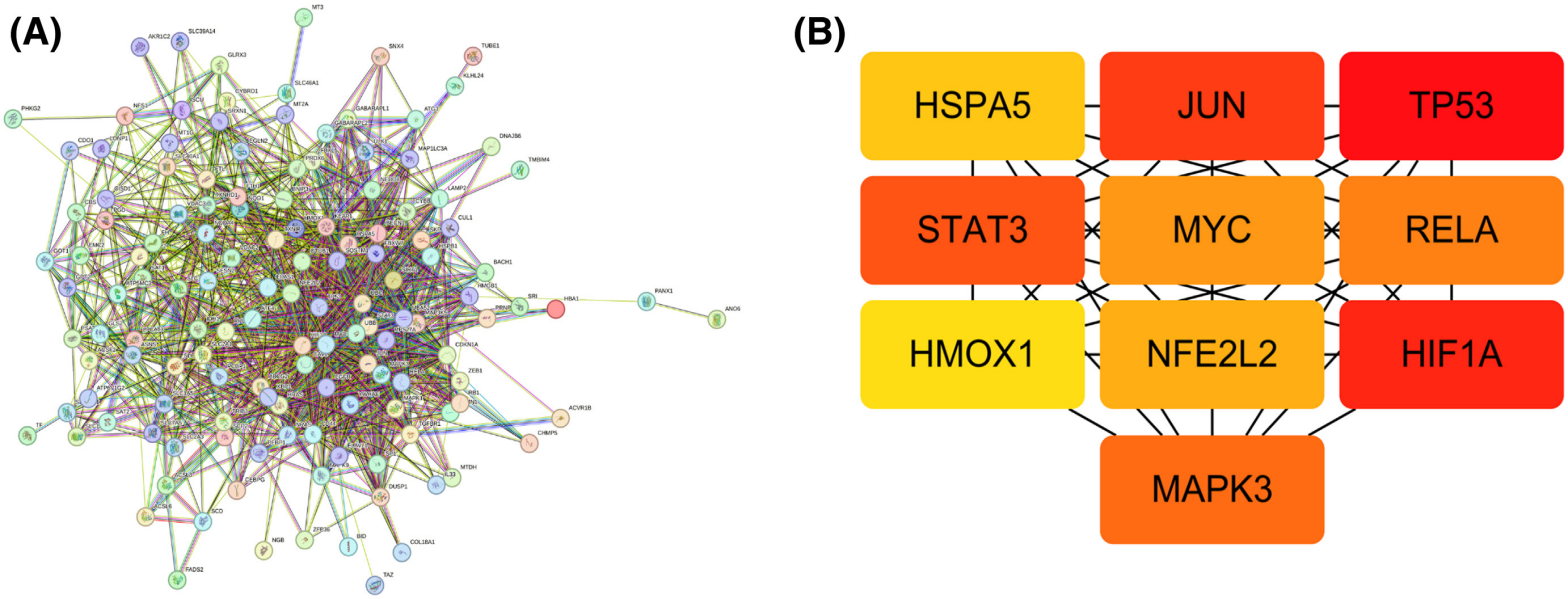
(A) PPI network involving 144 ferroptosis‐related DEGs between glioma and normal brain tissues. (B) The top 10 hub genes in the PPI network. The darkest node represents the highest MCC score.

**FIGURE 6:**
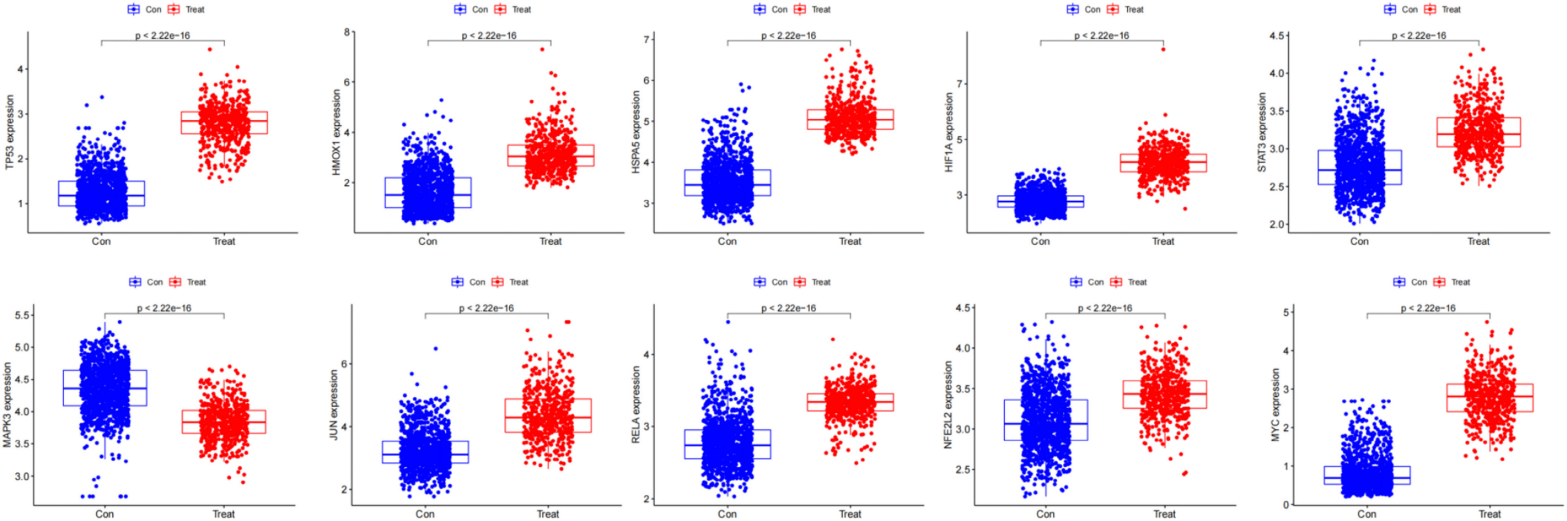
Expression levels of the top 10 hub genes in glioma (*n* = 534) and normal brain tissues (*n* = 1152).

Based on the intersections between 6298 DEGs and 296 ferroptosis‐related genes yielded in the FerrDb database, 144 ferroptosis‐related DEGs were identified (Figure 4), and imported into the STRING database to depict a PPI network (Figure 5A). Using the maximal clique centrality (MCC), the top 10 hub genes in the PPI network were screened, including *TP53*, *HIF1A*, *JUN*, *STAT3*, *MAPK3*, *RELA*, *MYC*, *NFE2L2*, *HSPA5* and *HMOX1* genes (Figure 5B). Compared with those of normal brain tissues, significantly upregulated TP53, HIF1A, JUN, STAT3, RELA, MYC, NFE2L2, HSPA5 and HMOX1, and downregulated MAPK3 were detected in glioma tissues (all *p* < 0.01, Figure 6).

### 3.4 Correlations between hub genes and m6A modification

**FIGURE 7:**
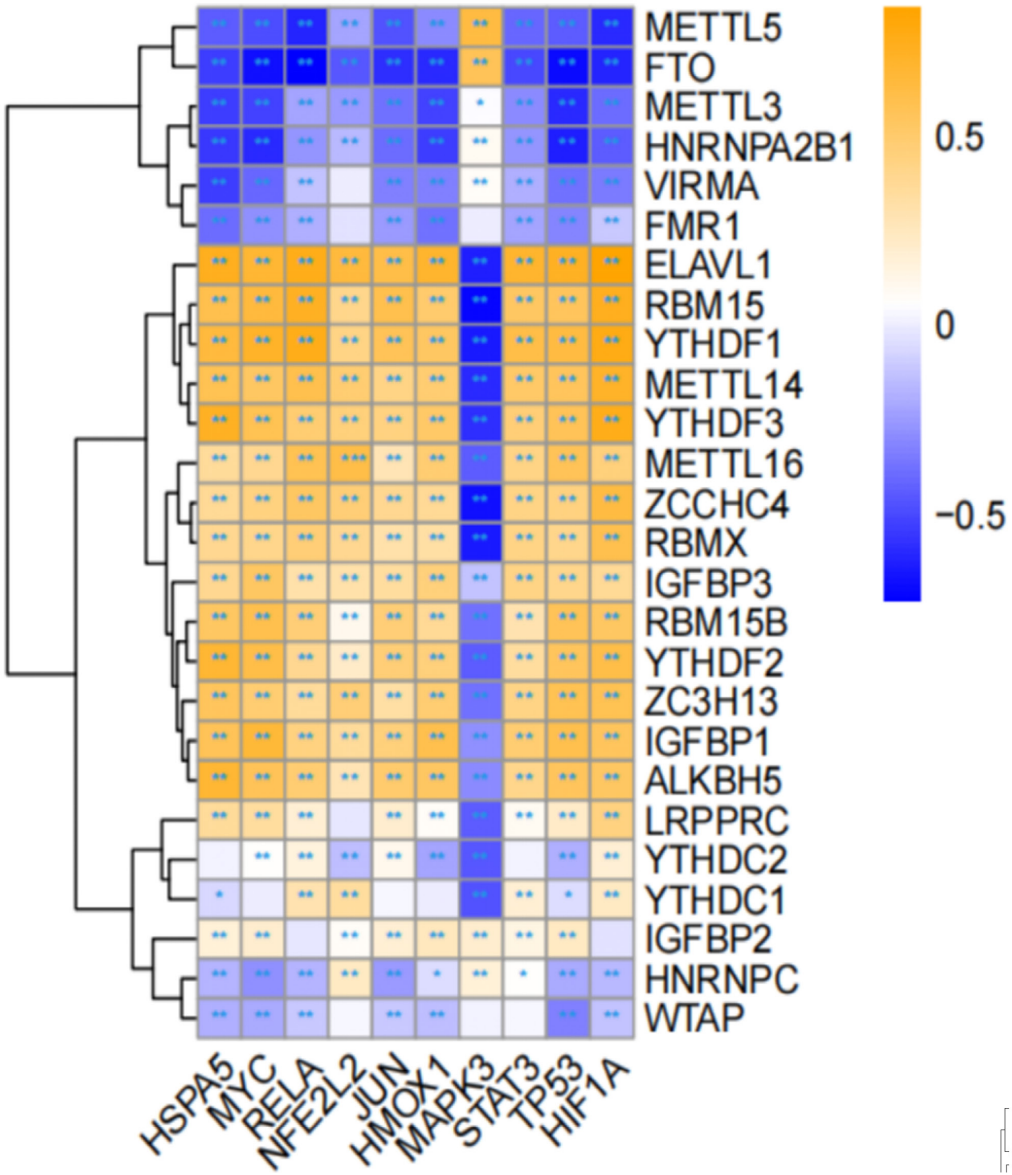
The Spearman correlation heat map visualising the correlation between the hub genes and m6A regulators. A colour gradient represents correlation transitions from blue (negative correlations) to white (no correlations) to yellow (positive correlations).

Spearman correlation between the top 10 hub genes and 26 common m6A regulators was analysed in R package. The strongest positive correlation was identified between METTL16 and NFE2L2 (Figure 7).

### 3.5 Expression levels of METTL16 and NFE2L2 in gliomas

**FIGURE 8:**
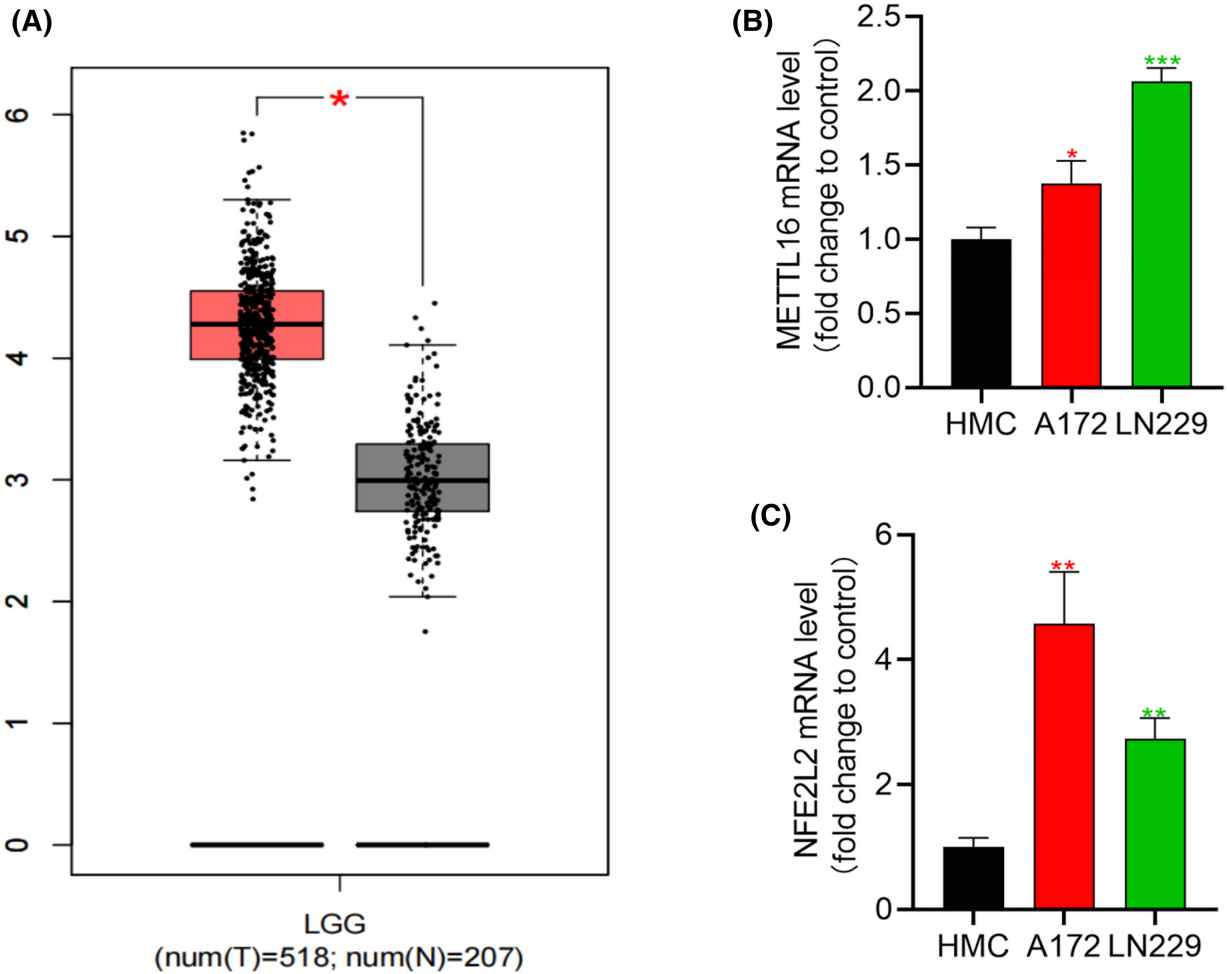
Expression levels of METTL16 and NFE2L2 in glioma tissues (A) and cell lines (*n* = 3/group) (B, C). *p < 0.05, **p < 0.01, ***p < 0.001.

In the UALCAN database, both METTL16 and NFE2L2 were found significantly upregulated in glioma tissues (Figure 8A). Consistently, they were significantly upregulated in the glioma cell lines A172 and LN229 than in those of the human meningeal cell line HMC (Figure 8B,C). LN229 cells were used in the following in vitro experiments due to the pronounced overexpression of METTL16 and NFE2L2.

### 3.6 METTL16 promotes the metastasis and inhibits ferroptosis of glioma cells

**FIGURE 9:**
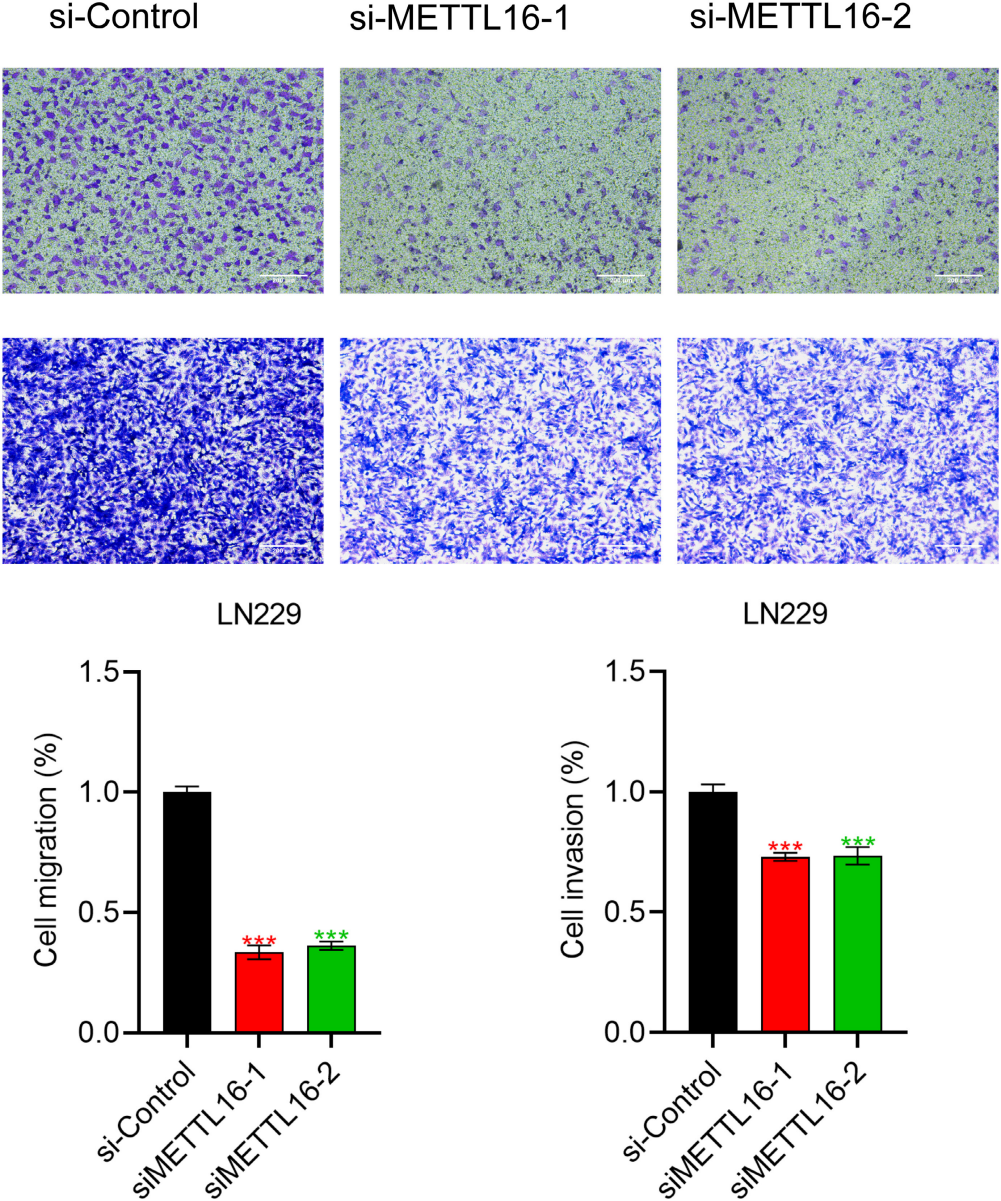
METTL16 promotes migration and invasion in glioma cells. LN229 cells were transfected with si‐Control, siMETTL16‐1 or siMETTL16‐2 for 48 h, followed by measurement of migratory and invasive cells via Transwell assay (*n* = 3/group) (magnification × 20, Scale bar 200 μm). ***p < 0.001.

**FIGURE 10:**
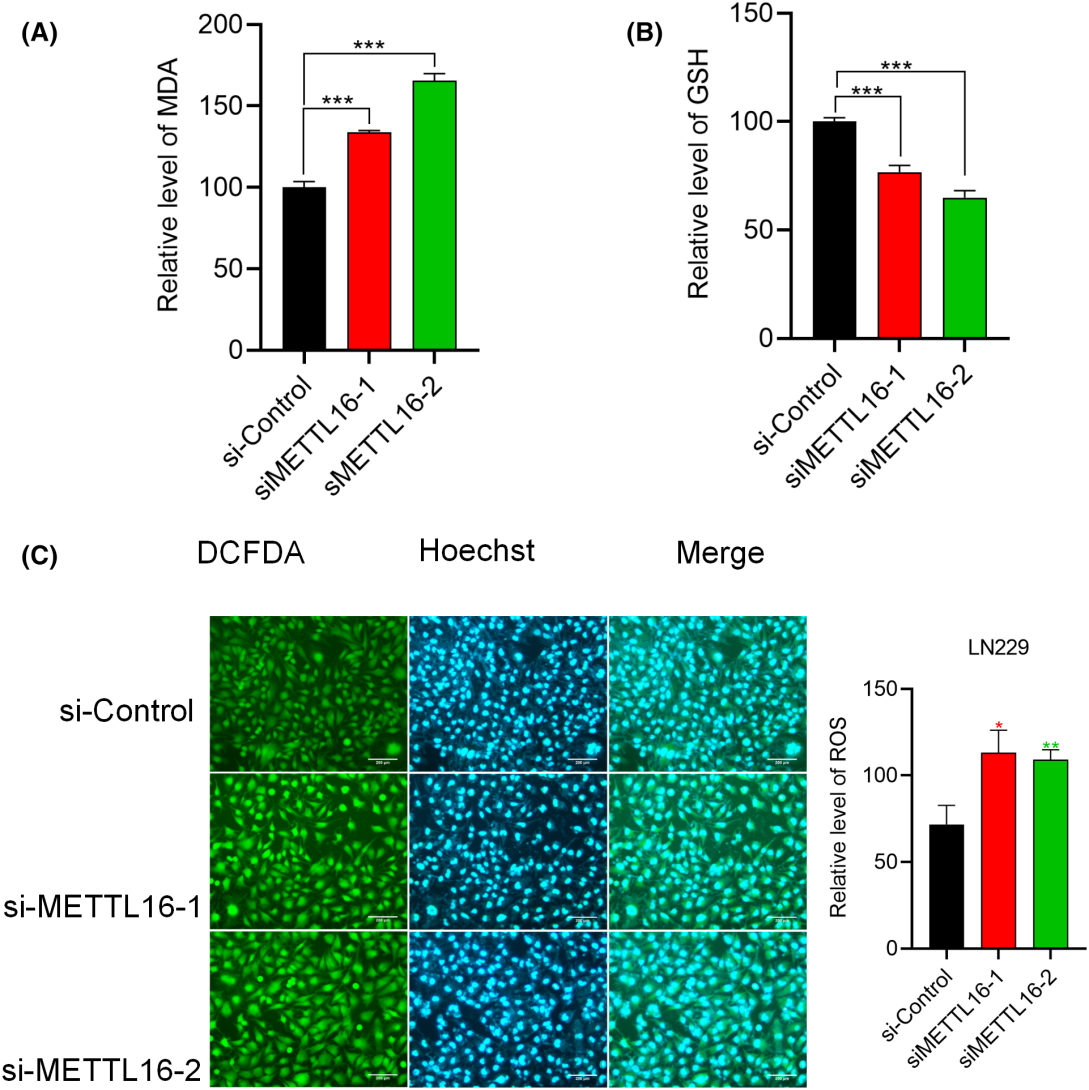
METTL16 inhibits ferroptosis in glioma cells. LN229 cells were transfected with si‐Control, siMETTL16‐1 or siMETTL16‐2 for 48 h, followed by measurements of MDA (A), GSH (B) and ROS levels (C) (*n* = 3/group) (magnification × 20, Scale bar 200 μm).*p < 0.05, **p < 0.01, ***p < 0.001.

We later examined the regulation of METTL16 on the behaviours of LN229 cells. Interestingly, knockdown of METTL16 significantly diminished migratory and invasive cells (Figure 9), suggesting that METTL16 promoted migration and invasion in glioma cells. In addition, knockdown of METTL16 significantly enhanced the content of MDA (Figure 10A) and ROS level (Figure 10C), but inhibited GSH level (Figure 10B), suggesting that METTL16 inhibited ferroptosis in glioma cells.

### 3.7 METTL16‐induced modification stabilizes NFE2L2 mRNA

**FIGURE 11:**
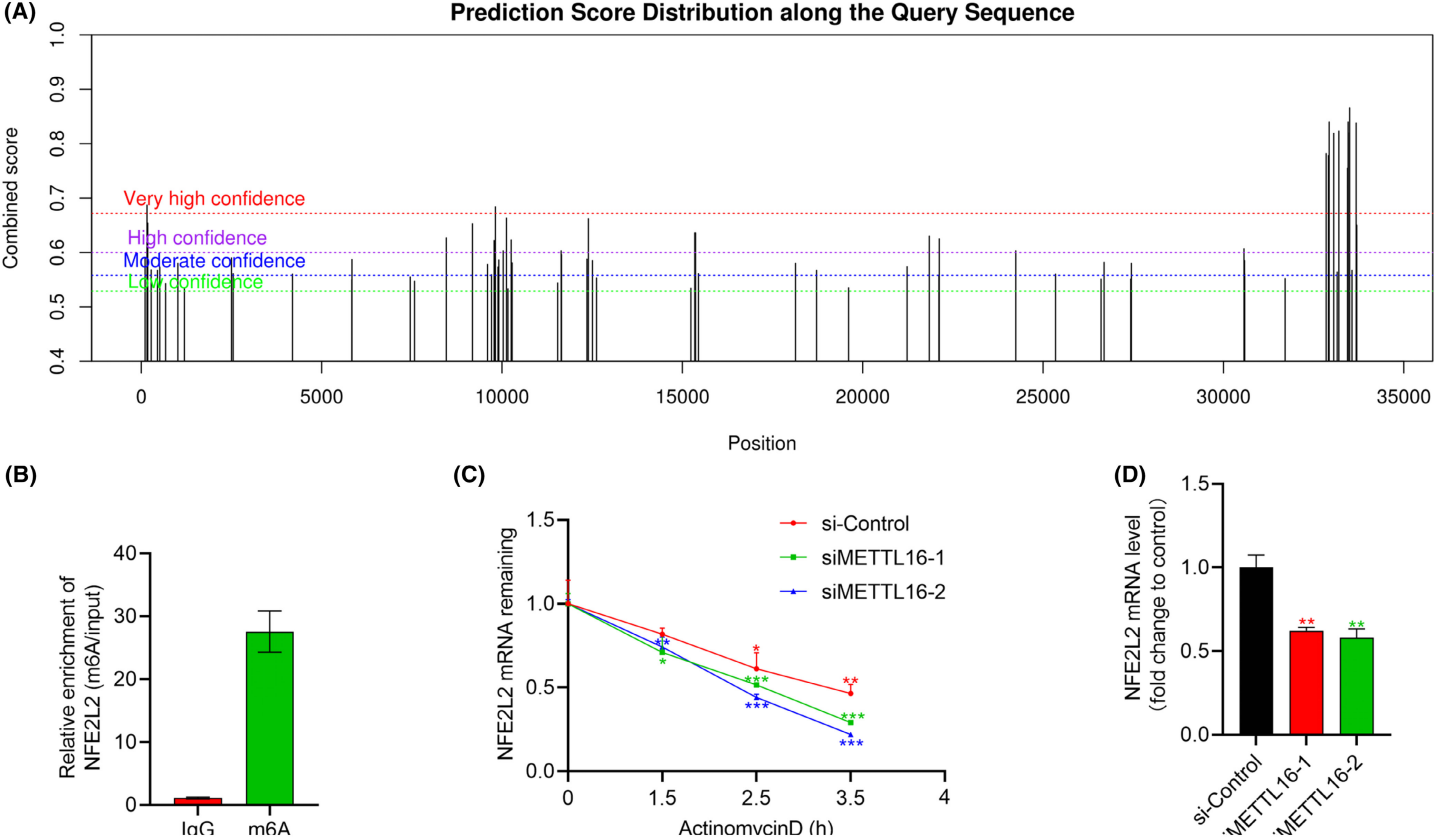
METTL16‐induced m6A modification stabilizes NFE2L2 mRNA. (A) m6A sites on the RNA sequences of NFE2L2 predicted by SRAMP. (B) The enrichment of NFE2L2 in the anti‐m6A antibody and IgG. (C, D) The mRNA levels of NFE2L2 in LN229 cells induced with actinomycin D (C) or not (D).(*n* = 3/group).*p < 0.05, **p < 0.01, ***p < 0.001.

Using the SRAMP prediction server, 43 m6A sites were predicted on the RNA sequences of NFE2L2 (Figure 11A). We later verified that NFE2L2 was significantly enriched in the anti‐m6A antibody by MeRIP‐qPCR (*p* < 0.05, Figure 11B). In actinomycin D‐induced LN229 cells, METTL16 knockdown evidently diminished the stability of NFE2L2 (*p* < 0.05, Figure 11C). The mRNA level of NFE2L2 was also significantly downregulated by transfection of siMETTL16‐1 and siMETTL16‐2 (*p* < 0.05, Figure 11D).

### 3.8 Tumour‐infiltrating immune cells in low‐grade gliomas

**FIGURE 12:**
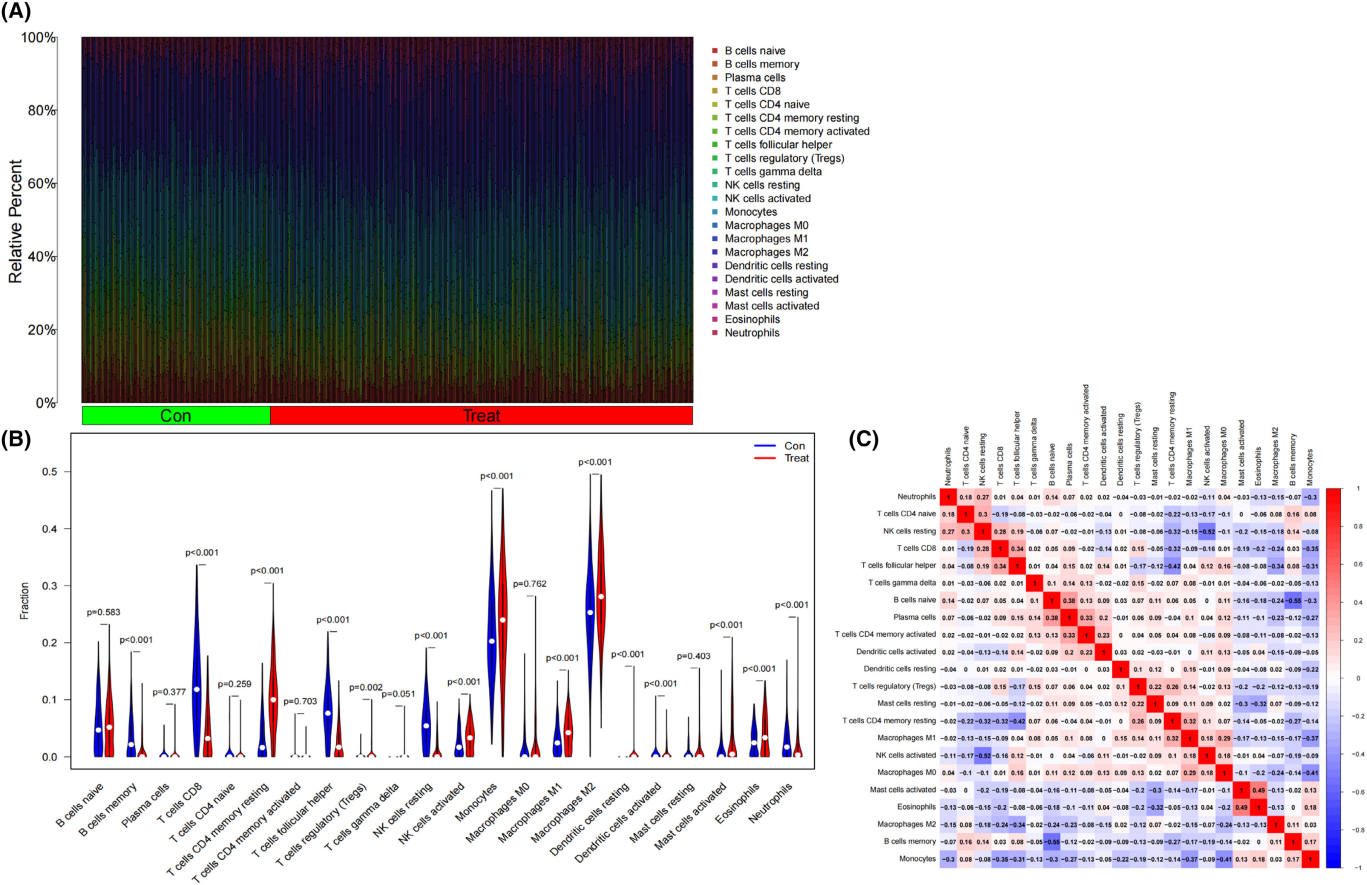
Tumour‐infiltrating immune cells in low‐grade gliomas. (A) Tumour‐infiltrating immune cells in low‐grade glioma tissues (red) and normal tissues (green). (B) Pearson correlation analysis of 22 types of tumour‐infiltrating immune cells in low‐grade gliomas. A colour gradient represents correlation transitions from blue (negative correlations) to red (positive correlations). (C) Violin plots visualising the proportions of 22 types of tumour‐infiltrating immune cells in low‐grade glioma tissues (red) and normal tissues (blue).

To further explore the correlations of METTL16 and NFE2L2 with the immune microenvironment in gliomas, we compared the abundances of 22 types of tumour‐infiltrating immune cells between low‐grade glioma and normal tissues using the CIBERSORT R script. There were evident differences in the abundances of CD8^+^ T lymphocytes, activated mast cells and M2 macrophages between low‐grade glioma and normal tissues (Figure 12A). Pearson correlation analysis demonstrated a notably positive correlation between the activated mast cells and eosinophils (*r* = 0.49, *p* < 0.05), and a negative correlation between memory B cells and naïve B cells (*r* = −0.55, *p* < 0.05) (Figure 12B,C).

### 3.9 Correlations of METTL16 and NFE2L2 with tumour‐infiltrating immune cells and immune checkpoints in low‐grade gliomas

**FIGURE 13:**
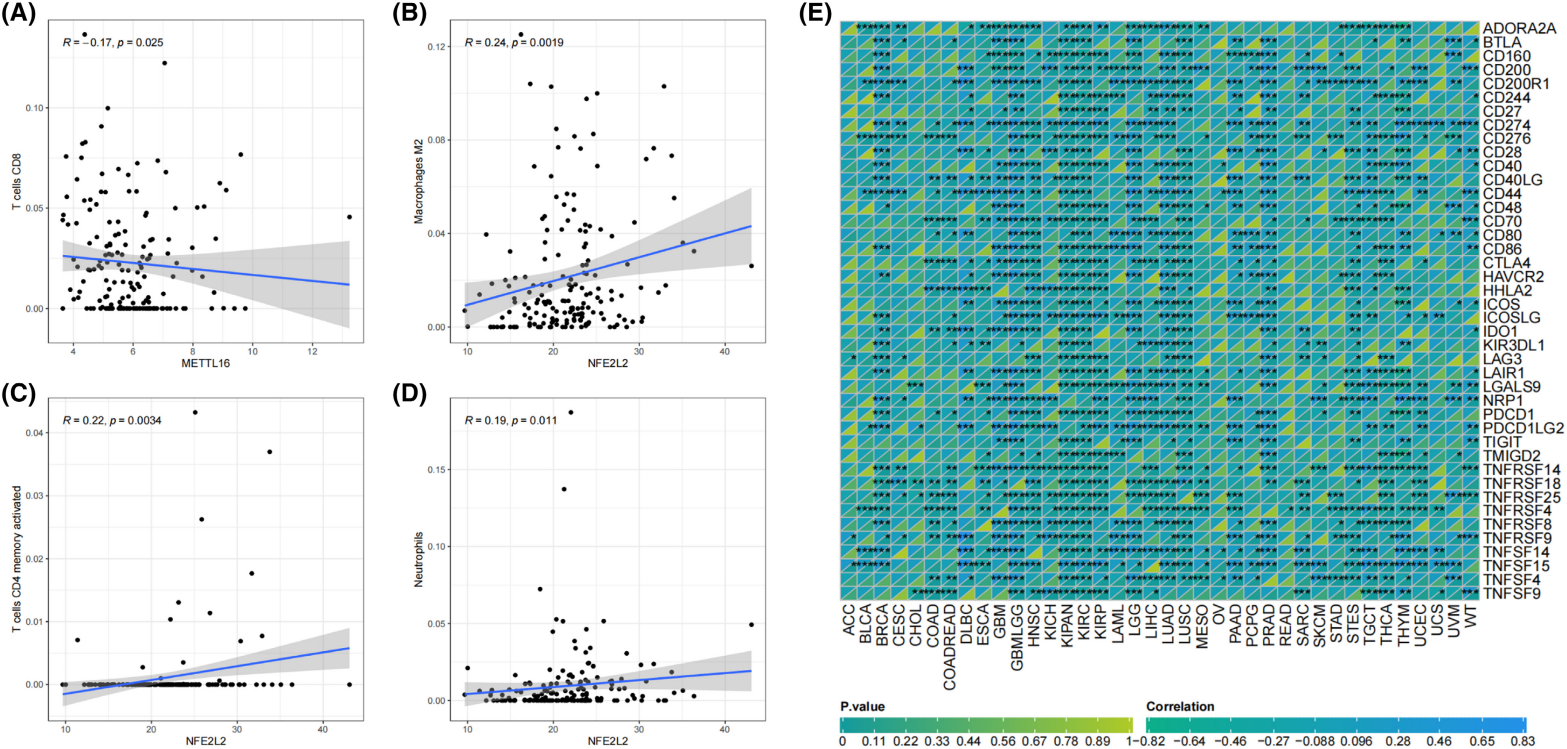
Correlations of METTL16 and NFE2L2 with the immune microenvironment in low‐grade gliomas. (A) A negative correlation of expression level of METTL16 with CD8^+^ T lymphocytes. (B‐D) Positive correlations of expression level of NFE2L2 with M2 macrophages (B), neutrophils (C) and activated memory CD4^+^ T cells (D). (E) Correlations of METTL16 and NFE2L2 with immune checkpoints in low‐grade gliomas.

In low‐grade gliomas, expression level of METTL16 had a negative correlation with that of CD8^+^ T lymphocytes (*r* = −0.17, *p* = 0.025, Figure 13A), and that of NFE2L2 had a positive correlation with M2 macrophages (*r* = 0.24, *p* = 0.0019, Figure 13B), neutrophils (*r* = 0.19, *p* = 0.011, Figure 13C) and activated memory CD4^+^ T cells (*r* = 0.22, *p* = 0.0034, Figure 13D). The expression level of NFE2L2 has a positive correlation with those of immune checkpoints TNFSF4, PDCD1, CD244 and ICOS (Figure 13E). The correlation of METTL16 with immune checkpoints in low‐grade gliomas, however, was not detectable.

## 4 DISCUSSION

Cancer has become one culprit of death worldwide, posing a significant public health challenge. Cancer development involves numerous signalling pathways and genes. Recent studies have indicated that the occurrence, progression, recurrence, and metastasis of gliomas are closely linked to m6A methylation modification, ferroptosis, and TME.^51^, ^52^ Thus, exploring the underlying mechanisms is essential.

M6A, one most prevalent RNA modification in mammalian cells, regulates RNA stability through mediating mRNA transcription, translation, splicing and degradation.^13^ Ferroptosis is an emerging type of programmed cell death, with typical characteristics of iron‐dependent generation of ROS and lipid peroxidation.^14^ It has been well concerned as a vital anti‐cancer mechanism.^53^ Rich evidence has shown the effect of m6A modification on tumour progression, metabolism, ferroptosis and immune microenvironment, indicating it as a regulator for immunotherapy.^54^ Xu et al.^55^ reported that METTL3 promotes the growth of lung adenocarcinomas and suppresses ferroptosis by stabilising SLC7A11 m6A modification. As a target of miR‐4443, METTL3 triggers ferroptosis in non‐small cell lung cancer by regulating the expression level of FSP1.^56^ ALKBH5 reduces the transcriptional level of SLC7A11 by deleting m6A modification, thereby promoting ferroptosis in colorectal cancer.^57^ The involvement of m6A‐related ferroptosis in gliomas, however, has not rarely reported.

Through a series of bioinformatic analyses, we first identified 6298 DEGs between glioma and normal tissues, which were mainly enriched in mRNA processing, ferroptosis pathways, pathways in cancers, etc. Intersecting between 296 ferroptosis‐related genes and 6298 DEGs, 144 ferroptosis‐related DEGs were identified and subjected to the PPI network. Then, the top 10 hub genes in the PPI network were yielded, and their correlations with 26 common m6A regulators were identified. Among them, the strongest positive correlation was found between METTL16 and NFE2L2.

METTL16, one of the m6A methyltransferases, is recognized as the second m6A methyltransferase discovered and extensively studied in human cancers.^58^ Its overexpression is correlated with a poor prognosis of liver cancer, colorectal cancer and breast cancer.^59^, ^60^, ^61^ A previous bioinformatics study suggested that METTL16 is involved in glioma genesis, but this finding has not been validated in further ex vivo experiments.^62^ Consistently, we also predicted that METTL16 was overexpressed in gliomas, which was later verified by qRT‐PCR. In breast cancer, METTL16 epigenetically upregulates GPX4 through m6A modification, thereby inhibiting ferroptosis and promoting cancer progression.^61^ In the present study, METTL16 knockdown prominently attenuated the migrative and invasive abilities of LN229 cells, and increased the content of MDA and ROS level, but inhibited GSH level, suggesting that METTL16 may be an oncogene that inhibits ferroptosis in glioma cells.

NFE2L2 (NFE2 like bZIP transcription factor 2) is a key transcription factor for antioxidant response. Under normal circumstances, Keap1 and NFE2L2 are interacted in the cytoplasm where Keap1 induces the ubiquitination and degradation of NFE2L2.^63^ Stimulated by oxidative stress, autophagy triggers the degradation of Keap1 and thus the release of NFE2L2. Free NFE2L2 moves into the nuclei to combine with antioxidant response elements, thus activating the GSH antioxidant system.^64^ The plasma membrane transporter xCT subsequently transports cysteine (Cys) from the extracellular space to the cytoplasm, which is a raw material to synthesize GSH. As a key ferroptosis biomarker, glutathione peroxidase 4 (GPX4) contributes to remove lipid ROS using GSH as a reducing agent.^65^ Excessive lipid ROS, however, results in ferroptosis by producing massive lipid peroxidation and damaging cell membrane. Overexpression of NFE2L2 is found to inhibit ferroptosis through eliminating ROS in cancer cells.^66^, ^67^ Primary grade IV brain tumour is accompanied with a poor survival, as NFE2L2 is upregulated. Moreover, fostered NFE2L2 expression increases resistance against ferroptosis.^68^ However, until now, there have been few studies on the role and mechanism of m6A in regulating NFE2L2 in glioma, and their potential regulatory effects await further investigation.

A total of 43 m6A sites were predicted on the RNA sequences of NFE2L2 using the online tool, and the correlation of NFE2L2 with m6A modification was later verified by MeRIP‐qPCR. In addition, knockdown of METTL16 significantly reduced the stability and mRNA level of NFE2L2, indicating the involvement of MELLT16 in the m6A modification of NFE2L2. This is consistent with previous experimental results.^69^

A new understanding about how the status of TME affects immune cell functions can provide new therapeutic opportunities for cancer immunotherapy.^70^ Both m6A and ferroptosis are potent regulators on immune cells in the TME.^71^, ^72^ The bioinformatic analysis revealed significant abundances in the proportions of CD8^+^ T lymphocytes, activated mast cells and M2 macrophages between low‐grade glioma and normal tissues. We noticed a positive correlation between the activated mast cells and eosinophils, and an opposite relationship between memory B cells and naïve B cells. The function of T lymphocytes, especially that of CD8^+^ T lymphocytes function, is suppressed, thus remarkably accelerating cancer progression.^73^ CD8 T cells can directly eliminate tumour cells by specifically targeting components of these cells or by secreting interferon‐gamma (IFN‐γ) and tumour necrosis factor‐beta (TNF‐β), thereby activating NK cells and macrophages to eradicate tumour cells.^74^ We therefore speculated that the infiltration of CD8^+^ T lymphocytes may realize the oncogenic role of METTL16 in low‐grade gliomas. This is consistent with previous experimental results.^75^ Tumour‐associated macrophages (TAMs) are a core group of immunosuppressive cells leading to immune evasion.^76^ TAMs are functionally divided into M1 and M2 macrophages.^77^ The former is the first‐line defence against microbial infections, which possesses a strong antigen presentation to induce Th1 response.^78^ M2 macrophages can repress immune responses to induce angiogenesis and tissue repair.^79^ As a result, M1 macrophages are tumour‐inhibiting factors and M2 macrophages exert the tumour‐promoting effect. Huang et al.^80^ reported that M2 macrophages are unfavourable to the prognosis of breast cancer, because that they reduce the efficacy of chemotherapy and radiotherapy via inhibiting the CD8^+^ T lymphocyte function. Hence, overexpressed NFE2L2 in low‐grade gliomas may stimulate cancer progression by increasing the infiltration of M2 macrophages. This is consistent with previous experimental results.^81^

Abnormal activation of immune checkpoints propels tumours to evade attacks from the immune system.^82^ We for the first time confirmed the positive correlation of NFE2L2 with immune checkpoints like TNFSF4, PDCD1, CD244 and ICOS, further verifying the regulatory effect of NFE2L2 on the immune system. Unfortunately, correlation of METTL16 with immune checkpoints in gliomas was not detected.

Taken together, we identified gene signatures involved in the m6A‐related ferroptosis in gliomas through an arsenal of bioinformatic analyses. NFE2L2 is strongly correlated with METTL16, both of which play critical roles in the immune response of low‐grade gliomas. However, we acknowledge several limitations in this study. First, this retrospective analysis using public databases might have introduced selection bias and potential confounding factors. TCGA and GTEx, known for their extensive, high‐quality datasets, were utilized in our study. This approach significantly enriches our understanding of disease mechanisms by providing a comprehensive view of gene expression in both diseased and normal tissues. Nevertheless, differences in sample processing and data normalisation between TCGA and GTEx might lead to batch effects. To address these, we employed the limma package in R, mitigating their impact on our findings. We believe that our analysis and conclusions are drawn with full consideration of these inherent limitations. Additionally, our experimental study using GBM cell lines had a small size, and it necessitates further larger cohort analysis and in vivo models. Third, the correlation of NFE2L2 with immune checkpoints in low‐grade gliomas should be explored to elucidate the exact mechanism. Our findings also warrant future validation in in vivo models. Lastly, the regulatory mechanisms of METTL16 and NFE2L2 in the TME remain to be elucidated.

Although challenged with those limitations, our study uncovers the oncogenic roles of METTL16 and NFE2L2 in GBM and paves the way for additional research in this domain. Through comprehensive analysis of gene expression data across various databases, we discovered significant upregulation of METTL16 and NFE2L2 in GBM tissues compared to normal brain samples. This finding positions them as potential biomarkers for disease prognosis assessment. In functional experiments using LN229 GBM cells, we observed that METTL16 influences cell migration, invasion and ferroptosis, reinforcing its potential as a therapeutic target. Further investigations, including MeRIP and actinomycin assays, affirmed the role of METTL16 in m6A modification of NFE2L2. Our research also ventured into examining the involvement of METTL16 and NFE2L2 in immune infiltration within the GBM microenvironment, uncovering correlations that suggest their impact on immune response modulation. Our research marks a significant stride in decoding GBM pathogenesis and highlights the potential of METTL16 and NFE2L2 as pivotal factors in disease progression and therapeutic innovation.

## 5 CONCLUSION

NFE2L2 is the hub gene involved in m6A‐related ferroptosis in gliomas, which is strongly correlated with METTL16. Both of NFE2L2 and METTL16 are involved in the immune response of low‐grade gliomas, serving as novel therapeutic targets for gliomas.

## AUTHOR CONTRIBUTIONS

**Yang Yang:** Writing – original draft (equal). **Liu Hao:** Data curation (equal). **Liu Guiyang:** Writing – review and editing (equal). **Piao Haozhe:** Writing – review and editing (equal).

## CONFLICT OF INTEREST STATEMENT

The authors have no conflict of interest to declare.

## Data Availability

The data that support the findings of this study are available in TCGA database (https://portal.gdc.cancer.gov) and GTEx project (http://xena.ucsc.edu/).
